# A comprehensive comparison of circulating tumor cells and breast imaging modalities as screening tools for breast cancer in Chinese women

**DOI:** 10.3389/fonc.2022.890248

**Published:** 2022-08-01

**Authors:** Xuan Shao, Xiaoyan Jin, Zhigang Chen, Zhigang Zhang, Wuzhen Chen, Jingxin Jiang, Zhen Wang, Ying Cui, Wan-Hung Fan, Ke Wang, Xiuyan Yu, Jian Huang

**Affiliations:** ^1^ Department of Breast Surgery, Second Affiliated Hospital, Zhejiang University School of Medicine, Hangzhou, China; ^2^ Key Laboratory of Tumor Microenvironment and Immune Therapy of Zhejiang Province, Hangzhou, China; ^3^ Department of Surgical Oncology, Taizhou Municipal Hospital, Taizhou, China; ^4^ Hangzhou Watson Biotech, Hangzhou, China

**Keywords:** breast cancer, circulating tumor cells, ultrasonography, mammography, magnetic resonance imaging, diagnosis

## Abstract

**Background:**

Circulating tumor cells (CTCs) have been recognized as a sensitive biomarker for breast cancer (BC). This study aimed to comprehensively compare CTC with imaging modalities, including ultrasonography, mammography, and contrast-enhanced magnetic resonance imaging (MRI) in screening for BC in Chinese women.

**Methods:**

Three hundred forty-three participants were enrolled in this study, including 102 treatment-naive BC patients, 177 with breast benign diseases (BBD) and 64 healthy female patients. All participants underwent CTC testing and at least one of the following examinations, ultrasonography, mammography, and MRI at the Second Affiliated Hospital of Zhejiang University between December 2017 and November 2020. CTCs were quantitatively assessed using cell counting (CTC detection rate/counts) and categorically examined using a cutoff value (CTC classification). The diagnostic power of CTC tests and imaging modalities, including accuracy and capability to predict clinicopathological characteristics of BC, were evaluated and compared.

**Results:**

CTC classification with a cutoff value of 2 showed a “good” diagnostic accuracy of 0.889 for early- to mid-stage BC comparable to breast imaging modalities using Breast Imaging-Reporting and Data System (BI-RADS). MRI demonstrated the highest sensitivity of 0.872 for BC, and CTC classification had the highest specificity of 0.938. A relatively low sensitivity was found for mammography in this cohort of patients. Successful detection of BC by CTC detection rate/counts, but not CTC classification, correlated with two important clinicopathological features, American Joint Committee on Cancer (AJCC) stage and tumor-node-metastasis (TNM) stage. The detection power of certain imaging modalities was also associated with AJCC stage (ultrasonography, *p* = 0.0438 and MRI, *p* = 0.0422) and lymph node metastasis (ultrasonography, 0.0157). There were clear correlations between CTC tests (counts or classification) and imaging BI-RADS scoring system in detecting positive BC cases (*p* < 0.05). Further correlation analysis suggested that CTC quantity, but not CTC classification, had the capability to predict clinicopathological traits of BC that were identified by ultrasonography.

**Conclusions:**

CTC tests have a diagnostic potency comparable to breast imaging modalities, and may be used as an alternative screening tool for BC.

## Background

Female breast cancer (BC) is a leading cause of global cancer incidence, with 2.3 million new cases reported globally in 2020 (1). Several risk factors that contribute to the elevated incidence of BC have been identified, including low birth rate, the postponement of childbearing, obesity, physical inactivity, and high prevalence of BC-related genetic mutations in women of certain heritage (1). High mortality rates of BC have been reported in some transitioning countries such as Melanesia and those in the sub-Saharan Africa region and has been at least partially attributed to the lack of effective and feasible population-based BC screening programs (1).

Breast palpation was the recommended first-line BC screening method in many countries (2), with evident limitations such as a low sensitivity, failure to improve the overall survival of BC patients, often causing mental health issues and resulting in over-treatment (2–4). Testing for serum tumor biomarkers, such as carcinoembryonic antigen (CEA), carbohydrate-containing protein antigen or cancer antigen 15-3 (CA15-3), CA125, and CA199, have also been adopted for BC screening (5). These tumor biomarkers are non-specific for BC and have a very low sensitivity and specificity for early-stage BC (5); they have been more commonly used to monitor cancer progression (6). Currently, the preferred BC screening methods are breast imaging modalities, including mammography, ultrasonography and magnetic resonance imaging (MRI) (7). Mammography has been recommended by the American Society of Clinical Oncology (ASCO) as it was the only imaging modality that improved the survival rate of BC patients (8). Mammography has a reported advantage in detecting early signs of BC, such as micro-calcification points (9). This method, however, was reported to be less sensitive for patients with dense breasts (10, 11), for example, women of Chinese descent  (12). Ultrasonography has been used as a more effective replacement in many areas in China (12). Similar to mammography, the sensitivity of ultrasonography depends on the breast tissue composition and structure (11, 13). Breast cancer diagnosis and treatment guidelines from the Chinese Society of Clinical Oncology (CSCO) suggested the combinational use of ultrasonography and mammography for a greater accuracy for Chinese women at a medium risk of BC (14). Breast MRI is the most sensitive diagnostic tool for many breast diseases, with a low risk of radiation exposure and providing images with high contrast and resolution. The wide application of breast MRI for BC screening, however, is hindered by its low cost-effectiveness; this modality is preferably chosen for the diagnosis of complicated BC cases and cancer staging. Apart from the cost, using breast imaging modalities for large-scale BC screening in many underdeveloped areas in China is impeded by the difficulty in accessing the service due to the lack of equipment, and a long waiting period.

Circulating tumor cells (CTCs) are tumor cells found in the human circulatory system, representing the precursors of metastases (15). Recent studies showed that the combinational use of CTC and breast imaging improved the diagnostic performance of ultrasonography and mammography for BC (16, 17). This study aimed to comprehensively examine and compare the diagnostic power of CTC tests and different imaging modalities, and to explore the potential of using CTC as an alternative of imaging modalities for the screening of BC in Chinese women.

## Methods

### Patients

One hundred and two patients with clinically diagnosed BC, 177 patients with benign breast disease (BBD), and 64 healthy female patients were recruited for this study; patients in the latter two categories were used as negative controls. All participants underwent pre-treatment CTC testing and at least one of the following imaging tests at the Second Affiliated Hospital of Zhejiang University between December 2017 and November 2020: ultrasonography, mammography, and MRI. Participants’ demographic information and clinicopathological characteristics of BC, including age, histological types and grades, hormone receptors, human epidermal growth factor receptor 2 (HER2), stage, and imaging Breast Imaging-Reporting and Data System (BI-RADS) results, were collected. The clinicopathological status of BC was confirmed by biopsy and histopathological examinations. This study followed the principles established in the Declaration of Helsinki and was approved by the ethics committee of the Second Affiliated Hospital, Zhejiang University School of Medicine (approval number, 2017-006). Written consent for participation in this study and publication of their case details were obtained from each participant.

### CTC tests

CTC detection and quantitation were carried out using CytoSorter^®^ (Hangzhou Watson Biotech, Hangzhou, China), a microfluidic-based immuno-capture CTC platform, and the method published by Jin etal. (16). In short, 4 ml of peripheral blood samples was diluted with the same volume of PBS, and aliquoted into two Leucosep^®^ tubes containing 2 ml of Histopaque^®^‐1077 (Sigma‐Aldrich) density gradient media. After density gradient centrifugation, the peripheral blood mononuclear cell (PBMC) layer was isolated and washed twice with 5% FBS DMEM. Cell pellet was re‐suspended in 190 μl of the same washing medium. CTCs were enriched and captured by the CytoSorter^®^ epithelial cell detection kit (Hangzhou Watson Biotech, Hangzhou, China), using CytoChipNano that has been pre-treated with EpCAM capture Ab for 1 h, as per the manufacturer’s protocol. Immunofluorescence staining was carried out for the CytoChipNano chip, using PanCK‐fluorescein isothiocyanate (FITC), CD45‐PE, and 4′,6‐diamidino‐2‐phenylindole (DAPI). The presence of CTC was qualitatively and quantitatively determined by an experienced technical staff. An Olympus BX61 microscope equipped with the CytoView™ software was used to scan CytoChipNano for potential CTCs, and a Nikon ECLIPSE Ti microscope was used to confirm CTC staining and localization. CTCs were defined as PanCK‐FITC^+^, CD45‐PE^‐^, and DAPI^+^ cells. CTC testing results were presented quantitatively as detection rate (number of patients with CTC detected/number of total participants) or cell counts (CTC counts), and categorically (CTC classification) as CTC ≥ cutoff value (cancer-positive) or CTC< cutoff value (cancer-negative).

### Breast imaging tests

Ultrasonography was carried out with an IU Elite^®^ (Philips Healthcare, Best, Netherlands). Mammography was performed using Selenia^®^ Dimensions (Hologic, Santiago, USA). Breast MRI was conducted on a Discovery^®^ MR750W (GE Healthcare, Illinois, USA). All imaging results were analyzed by two qualified radiologists, using criteria from the American College of Radiology (ACR) BI-RADS or following the Society of Nuclear Medicine operative guidelines. ACR BI-RADS was used for risk assessment of likelihoods of BC, with scores/categories of 1–6 representing negative, benign, probably benign, suspicious or indeterminate abnormality, highly suggestive of malignancy, and known cancer, respectively. Category 4 can be further divided into three sub-categories: 4A, 4B, and 4C for a low, moderate, and high suspicion of malignancy (18). Breast tissue composition and tumor size were visually estimated according to BI-RADS classification (18).

### Statistical analysis

Statistical analyses were performed using SPSS 20 (IBM, Armonk, NY, USA). Student’s *t*-test was used to compare continuous variables and the *χ* ^2^ test and Fisher’s exact test were chosen for the comparison of categorical parameters. One-way analysis of variance (ANOVA) was carried out for differences among multiple groups. The cutoff values of CTC and imaging BI-RADS for BC diagnosis were determined using the highest Youden index (sensitivity + specificity − 1). CTC counts or imaging BI-RADS ≥ cutoff values were defined as BC-positive. The receiver operating characteristic (ROC) curves were plotted to evaluate the diagnostic performance parameters, such as sensitivity, specificity, accuracy, and area under the curve (AUC). A *p*-value less than 0.05 was considered statistically significant.

## Results

### Clinical characteristics of participants

Clinical and laboratory data of all 343 participants were used for analysis. Demographic, clinical, and pathological characteristics of all participants are summarized in **Supplementary Table 1**. No significant difference in age was found between the BC group (median age, 53.7 years; range, 29–75 years) and the negative control group (median age, 43.2 years; range, 22–73 years). Among 102 BC patients, 101 underwent ultrasonography, 84 had mammography, and 91 received MRI. In the negative control group, 240 underwent ultrasonography, 106 had mammography, and 6 received MRI. Most patients in the BC group had early-mid stage cancer confirmed by a biopsy and histopathological analysis, with 42 having AJCC stage I BC, 47 with stage II BC, and 13 with stage III BC; no stage IV cases were found in the BC group.

### Comparing diagnostic performance of CTC tests and imaging BI-RADS for breast cancer

The detection rates (and average counts) of CTC for healthy volunteers, and patients with BBD and BC were 17.2% (0.2), 40.7% (0.5), and 91.2% (2.5), respectively. For participants with clinically diagnosed BC, CTC detection rates (and average counts) in stage I–III BC patients were 92.9% (2.1), 87.2% (2.4), and 100% (4.2), respectively.

We compared the diagnostic performance of CTC classification and the well-established BI-RADS of different imaging modalities. The highest Youden index of 0.712, 0.692, 0.600, and 0.705 were achieved for CTC, ultrasonography, mammography, and MRI, respectively, and the cutoff values of CTC and imaging BI-RADS were set to 2 and 4B (**Table 1**). MRI had the highest sensitivity of 0.872, followed by ultrasonography (0.842), CTC classification (0.775), and mammography (0.694). CTC classification had the highest specificity of 0.938, followed by mammography (0.906), ultrasonography (0.850), and MRI (0.833). AUCs of CTC classification, ultrasonography BI-RADS, mammography BI-RADS, and MRI BI-RADS were 0.856, 0.854, 0.810, and 0.843, respectively, suggesting good accuracy for all these tests. CTC classification had the highest accuracy of 0.889 for BC diagnosis.

**Table 1:** Diagnostic performance of CTC classification and imaging modalities for BC at different cutoff values.

Cutoff value^1^	Sensitivity	Specificity	Youden Index	Accuracy	AUC
CTC classification (*n* = 102 + 241)**^2^**					
1	0.912	0.656	0.567	0.732	0.784
2	0.775	0.938	0.712	0.889	0.856
3	0.314	0.988	0.301	0.787	0.651
US BI-RADS ^3^ (*n* = 101 + 240)					
4A	0.960	0.621	0.581	0.721	0.791
4B	0.842	0.850	0.692	0.848	0.854
4C	0.594	0.992	0.586	0.874	0.791
MG BI-RADS (*n* = 72 + 106)					
4A	0.806	0.736	0.541	0.765	0.771
4B	0.694	0.906	0.600	0.820	0.810
4C	0.472	0.981	0.453	0.775	0.727
MRI BI-RADS (*n* = 86 + 6)					
4A	0.930	0.167	0.097	0.880	0.549
4B	0.872	0.833	0.705	0.870	0.843
4C	0.698	0.833	0.531	0.707	0.758

^1^Cutoff values are determined by the highest Youden index.

^2^n = breast cancer positive cases + negative cases

^3^Imaging BI-RADS scores indicate different levels of likelihood of breast cancer: 4A, low (2% < likelihood ≤10%); 4B, moderate (10% < likelihood ≤ 50%); and 4C, high (50% < likelihood ≤ 95%).

CTCs, circulating tumor cells; BI-RADS, breast imaging-reporting and data system; BC, breast cancer; AUC, area under receiver operating characteristic curve; n, number of participants (BC patients + negative controls); US, ultrasonography; MG, mammography; MRI, magnetic resonance imaging.

### Correlations between CTC tests, imaging BI-RADS and clinicopathological status of the cancer

CTC detection rate/counts, CTC classification, ultrasonography, mammography, and MRI were all able to distinguish BC patients from control participants (*p* < 0.001, **Table 2**). We sought to determine whether the capabilities of CTC tests and imaging BI-RADS to detect BC were correlated to clinicopathological status of the cancer, such as different AJCC stages (0–IV), different TNM stages (tumor stages Tis, T1, T2, T3, and T4, and nodal stages N0, N1, N2, and N3), with or without lymph node metastasis, and different molecular subtypes. CTC classification and mammography BI-RADS at the pre-determined cutoff values appeared to be irrelevant to any of the above-mentioned clinicopathological parameters (*p* > 0.05). The successful detection of BC by ultrasonography BI-RADS was significantly correlated with cancer stage and lymph node metastasis (*p* = 0.0438 and 0.0157, respectively), while that of MRI was only correlated with cancer stage (*p* = 0.042). CTC detection rates/counts were correlated with AJCC stages (*p* = 0.0084) and TNM T stage (*p* = 0.0301), but not with lymph node metastasis or molecular subtypes (*p* > 0.05). Such correlation suggested that CTC detection rates/counts, and ultrasonography BI-RADS and MRI BI-RADS results might be used as predictors for certain clinicopathological changes of BC.

**Table 2:** Correlation of the detection power of CTC tests and imaging modalities and BC clinicopathology^*^.

Characteristics	*p*-value				
	CTC detection/counts(*n* = 102)	CTC classification(*n*= 102)	US (*n* = 101)	MG (*n* = 72)	MRI (*n* = 76)
BC versus Control	**<0.0001**	**< 0.0001**	**< 0.0001**	**< 0.0001**	**0.0008**
AJCC Stage	**0.0084**	0.0964	**0.0438**	0.3332	**0.0422**
TNM T Stage	**0.0301**	0.2417	0.1923	0.1668	0.116
TNM N Stage	0.0712	0.3921	0.0865	0.6971	0.422
Lymph Node Metastasis	0.1673	0.3269	**0.0157**	0.2609	0.0747
Molecular Subtypes	0.8058	0.4713	0.9188	0.8881	0.088

*Cutoff values were set to 2 and 4B for CTC classification and imaging BI-RADS, respectively, to achieve the best diagnostic performance for individual methods.

Numbers in bold are statistically significant.

CTCs, circulating tumor cells; BI-RADS, breast imaging-reporting and data system; BC, breast cancer; n, number of patients; US, ultrasonography; MG, mammography; MRI, magnetic resonance imaging; AJCC, American Joint Committee on Cancer; TNM, tumor-node-metastasis.

### Paired comparison of CTC tests with ultrasonography

We further examined whether CTC tests correlated with individual imaging modalities in correctly detecting BC and delivering information useful for cancer staging. CTC detection rate/counts and CTC classification were compared with imaging BI-RADS scores for BC detection, and with BI-RADS-suggested tumor size and breast tissue composition categories for cancer staging. A significant correlation was found between both CTC detection/counts and classification and ultrasonography BI-RADS (**Table 3**); 91.5% of participants who were negative for BC in ultrasonography (with BI-RADS ≤ 4A) had CTC < 2, while 85.7% of those suspicious of BC in ultrasonography (with BI-RADS ≥ 4C) had CTC ≥ 2. Participants with BI-RADS ≤ 4A and ≥ 4B had average CTC counts of 0.5 and 2.1 respectively. For patients who had tumor sizes estimated by ultrasonography, CTC detection/counts showed a significant correlation with tumor sizes grouped into 1a + 1b (T ≤ 1 cm), 1c (1 cm < T ≤ 2 cm), 2 (2 cm < T ≤ 5 cm), and 3 (T > 5 cm) (*p* = 0.0309), with higher CTC counts found in patients with bigger tumors. No correlation was found between tumor size detected by ultrasonography and CTC classification (*p* > 0.05).

**Table 3:** Correlations of CTC testing and ultrasonography in BC diagnosis.

Group	*n*	CTC Detected	CTC Detection Rate (%)	Average CTC Count (Range)	*p*	CTCs ≥ 2	CTCs < 2	*p*
***US BI-RADS*** (*n* = 101 + 240)^*^								
1	64	12	18.8	0.2 (0–1)	**<0.0001**	0	64	**<0.0001**
2	7	3	42.9	0.4 (0–1)		0	7	
3	82	25	30.5	0.4 (0–4)		7	75	
4A	71	41	57.7	0.8 (0–6)		12	59	
4B	54	36	66.7	1.1 (0–3)		21	33	
4C	31	27	87.1	2.9 (0–15)		25	6	
5	31	29	93.5	2.8 (0–9)		28	3	
6	1	1	100	2		1	0	
≦ 4A	224	81	36.2	0.5 (0–6)	**<0.0001**	19	205	**<0.0001**
≧ 4B	117	93	79.5	2.1 (0–15)		75	42	
***US Tumor Size*** (*n* = 101)^#^								
1a	3	3	100	1.7 (1–2)	0.0639	2	1	0.5181
1b	8	7	87.5	1.6 (0–3)		5	3	
1c	38	36	94.7	2.3 (0–7)		32	6	
2	49	43	87.8	2.8 (0–15)		37	12	
3	3	3	100	4		3	0	
1a+1b	11	10	90.9	1.6 (0–3)	**0.0309**	7	4	0.3589
1c	38	36	94.7	2.3 (0–7)		32	6	
2	49	43	87.8	2.8 (0–15)		37	12	
3	3	3	100	4		3	0	

^*^US BI-RADS score of 0 indicates an incomplete test. Scores 1–6 represent negative, benign, probably benign, suspicious or indeterminate abnormality, highly suggestive of malignancy, and known cancer, respectively. Score 4 is subdivided into three subscores: 4A, 4B, and 4C for a low, moderate, and high suspicion of malignancy, respectively.

^#^Tumor size T categories, 1a, 1b, 1c, 2, and 3 for BC of T ≤ 0.5 cm, 0.5 cm < T ≤ 1 cm, 1 cm < T ≤ 2 cm, 2 cm < T ≤ 5 cm, and T > 5 cm, respectively.

Numbers in bold are statistically significant.

CTCs, circulating tumor cells; BC, breast cancer; n, number of patients; US, ultrasonography; BI-RADS, breast imaging reporting and data system.

### Paired comparison of CTC tests with mammography

A significant correlation was found between CTC detection rate/counts and classification and mammography BI-RADS scores in diagnosing BC (**Table 4**); 82.2% of participants considered to be BC-negative (with BI-RADS ≦ 4A) had CTC < 2, while 77.8% of those considered to have suspicious BC (with BI-RADS ≧ 4C) had CTC ≧ 2. Participants with BI-RADS score ≦ 4A and ≧ 4B had average CTC counts of 0.7 and 2.4, respectively. No significant correlation was found between CTC detection rate/counts or classification and tumor sizes or breast composition suggested by mammography BI-RADS.

**Table 4:** Correlations of CTC testing and mammography in BC diagnosis.

Group	*n*	CTC Detected	CTC Detection Rate (%)	Average CTCs Count (Range)	*p*	CTCs ≥ 2	CTCs < 2	*p*
***MG BI-RADS*** (*n* = 72 + 106)^§^								
1	6	2	33.3	0.4 (0–1)	**<0.0001**	0	6	**<0.0001**
2	34	11	32.4	0.4 (0–3)		3	31	
3	52	29	55.8	0.9 (0–3)		13	39	
4a	26	15	57.7	0.9 (0–4)		5	21	
4b	24	18	75.0	2.3 (0–15)		15	9	
4c	31	28	90.3	2.5 (0–9)		24	7	
5	5	5	100.0	2.2 (1–3)		4	1	
≦ 4a	118	57	86.4	0.7 (0–4)	**<0.0001**	21	97	**<0.0001**
≧ 4b	60	51	94	2.4 (0–15)		43	17	
***MG Tumor Size*** (*n* = 43)^*^								
1a	1	1	100	3	0.2906	1	0	0.5951
1b	5	3	60	1.4 (0–3)		3	2	
1c	13	12	92.3	2.4 (0–6)		11	2	
2	22	20	90.9	2.2 (0–9)		14	8	
3	2	1	50	2 (0–4)		1	1	
***MG Breast Tissue Composition Categories*** (n = 82)^#^								
a	8	8	100	2.4 (1–4)	0.6793	6	2	0.4119
b	12	11	91.7	2.6 (0–9)		9	3	
c	52	47	90.4	2.6 (0–15)		42	10	
d	10	10	100	3.1 (2–7)		10	0	
a + b	20	19	95	2.5 (0–9)	0.8808	17	5	0.5244
c + d	62	57	91.9	2.7 (0–15)		52	10	

^§^MG BI-RADS scores 1–6 represent negative, benign, probably benign, suspicious or indeterminate abnormality, highly suggestive of malignancy, and known cancer, respectively. Score 4 is subdivided into three subscores: 4A, 4B, and 4C for a low, moderate, and high suspicion of malignancy, respectively.

^*^Tumor size T 1a, 1b, 1c, 2, and 3 represent BC of T ≤ 0.5 cm, 0.5 cm < T ≤ 1 cm, 1 cm < T ≤ 2 cm, 2 cm < T ≤ 5 cm, and T > 5 cm, respectively.

^#^Breast composition category a = almost entirely fat; category b = scattered fibroglandular densities; category c = heterogeneously dense; and category d = extremely dense.

Numbers in bold are statistically significant.

CTCs, circulating tumor cells; BC, breast cancer; n, number; MG, mammography; BI-RADS, breast imaging reporting and data system.

### Paired comparison of CTC testing with MRI

There were significant correlations between CTC detection rate/counts, CTC classification, and MRI BI-RADS scores in diagnosing BC (**Table 5**, *p* = 0.0267 and 0.0123, respectively); 62.5% of participants considered to be BC-negative (with BI-RADS ≦ 4A) had CTC < 2, while 80.3% of those who had suspicious BC (with BI-RADS ≧ 4C) had CTC ≧ 2. Participants with BI-RADS score ≦ 4A and ≧ 4B had average CTC counts of 1.4 and 2.5, respectively. No significant correlation was found between CTC detection rate/counts or CTC classification and vital clinicopathological parameters detected by MRI, including tumor sizes, breast tissue composition, and MRI time–signal intensity curves (TIC) indicative of the malignancy of tumor.

**Table 5:** Correlations of CTC tests and magnetic resonance imaging examination in BC diagnosis.

Group	*n*	CTC Detected	CTC Detection Rate (%)	Average CTCs Count (Range)	*p*	CTCs ≥ 2	CTCs < 2	*p*
***MR BI-RADS*** (*n* = 86 + 6)^§^								
3	7	6	85.7	2.1 (0–6)	**0.0267**	4	3	**0.0123**
4a	9	6	67	0.9 (0–2)		2	7	
4b	15	15	100	2.2 (1–7)		11	4	
4c	31	27	87	2.7 (0–15)		24	7	
5	15	15	100	2.5 (1–5)		14	1	
6	15	13	86.7	2.4 (0–5)		11	4	
≦ 4a	16	9	81.8	1.4 (0–6)	**0.0038**	6	10	**0.0018**
≧ 4b	76	70	93.3	2.5 (0–15)		60	16	
***MR Tumor Size*** (*n* = 83)^*^								
1a	2	2	100	1.5 (1–2)	0.1843	1	1	0.2827
1b	4	3	75	2.8 (0–6)		3	1	
1c	36	31	86.1	1.8 (0–4)		26	10	
2	35	33	94.3	2.6 (0–9)		30	5	
3	6	6	100	2.2 (1–4)		3	3	
***MR Breast Tissue Composition Categories*** (*n* = 91)^#^								
a	3	1	33.3	2.4 (1–4)	0.683	1	2	0.1837
b	27	24	88.9	2.6 (0–9)		19	8	
c	52	49	94.2	2.6 (0–15)		43	9	
d	9	9	100	3.1 (2–7)		7	2	
a + b	30	25	83.3	2.5 (0–9)	0.4016	20	10	0.1034
c + d	61	58	95.1	2.7 (0–15)		50	11	
***MR TIC*** (*n* = 91)^¥^								
I	22	20	90.9	2.7 (0–15)	0.84	16	6
II	38	36	94.7	2.3 (0–7)		31	7
III	27	23	85.2	2.5 (0–9)		21	6

^§^BI-RADS scores 1–6 represent negative, benign, probably benign, suspicious or indeterminate abnormality, highly suggestive of malignancy, and known cancer, respectively. Score 4 is subdivided into three subscores: 4A, 4B, and 4C for a low, moderate, and high suspicion of malignancy, respectively.

^*^Categories of tumor size T 1a, 1b, 1c, 2, and 3 represent BC with T ≤ 0.5 cm, 0.5 cm < T ≤ 1 cm, 1 cm < T ≤ 2 cm, 2 cm < T ≤ 5 cm, and T > 5 cm, respectively.

^#^Breast composition categories a = almost entirely fat; category b = scattered fibroglandular densities; category c = heterogeneously dense; and category d = extremely dense.

^¥^TIC: I = progressive or persistent enhancement pattern, usually considered benign with only a small proportion (~9%) of malignant lesions having this pattern; II = plateau pattern, considered concerning for malignancy; III = washout pattern, considered strongly suggestive of malignancy.

Numbers in bold are statistically significant.

CTCs, circulating tumor cells; BC, breast cancer; n, number of patients; MR, magnetic resonance imaging; BI-RADS, breast imaging reporting and data system; TIC, time-signal intensity curves.

## Discussion

Breast cancer remains one of the most common malignant tumors with high mortality in China (12). Early detection and intervention are key strategies to reduce BC-related death. The aims of this study were to comprehensively compare the diagnostic performances of CTC tests and widely used breast imaging modalities and to explore the potential of using CTC as an alternative for BC screening. Key findings of this study include the following: (1) CTC tests had high diagnostic accuracy comparable to that of breast imaging modalities for early and mid-stage BC, (2) CTC tests correlated with imaging BI-RADS scoring system in detecting BC, and (3) no correlation was found between CTC classification and clinicopathological status of BC identified by imaging modalities, while CTC detection rate and counts appeared to correlate with cancer stage and tumor size estimated by ultrasonography.

This work suffered from its single-center nature that only allowed us to recruit small and unparalleled numbers of patients and healthy volunteers who were willing to participate in our study within the predefined period. We also noticed a low specificity and Youden index associated with MRI at the cutoff of 4A in diagnosing BC (**Table 1**). This was probably due to the very small number of negative controls recruited for this specific test, which was another evident limitation of the current study. In this prospectively designed study, all enrolled subjects were allowed to choose one, two, or three imaging modalities to encourage their participation. This design unexpectedly led to small sample sizes of negative controls for MRI and mammography. Increasing participant number and encouraging participants to undergo all imaging modalities may address this limitation and allow for a more accurate comparison between CTC and imaging modalities in detecting early to mid-stage BC. A large multicenter prospective cohort study should be further carried out to validate our findings.

Although mammography is the ASCO-recommended test for BC screening, it is less preferred for Chinese women due to its low sensitivity for patients with dense breasts (10, 11). Ultrasonography has been widely used as an alternative or combinational tool for BC screening in China. The wide use of mammography, ultrasonography, and MRI for BC screening in China and many other developing countries, however, is still hindered by the difficulty of patients in accessing required equipment and service, and a long waiting period. Leaving blood samples in a local collection center for further CTC analysis in a qualified pathology laboratory will significantly minimize patients’ efforts and increase the acceptance of general public for BC screening.

We explored the potential of using CTC as an alternative test for BC screening. Others reported that CTC tests could be used as a diagnostic aid for BC, supplementary to breast imaging (16, 17). We found a significant correlation between CTC detection rate/counts, CTC classification and the BI-RADS scoring system of different imaging modalities in detecting positive BC cases. In the context of overall diagnostic accuracy, CTC classification at a pre-determined cutoff value of 2 showed the highest specificity and a “good” diagnostic performance for early- to mid-stage BC, comparable to three widely used breast imaging modalities. This implicated a practical applicability of CTC in BC screening and diagnosis. A recent study by Jin etal. (16) also reported similarly high detection rates of early to mid-stage BC by CTC (16). Ultrasonography demonstrated a high sensitivity and a relatively low specificity, in agreement with what has been reported elsewhere (19). Unsurprisingly, mammography showed a relatively lower sensitivity for this study cohort of Chinese patients (10–12). MRI had the highest sensitivity among all examined tests. Due to its low cost-effectiveness as a screening test, MRI has been mostly used for BC staging, problematic diagnosis when a direct biopsy is infeasible, or monitoring the outcome of primary systemic therapies (20).

We also speculated that CTC might be informative in predicting clinicopathological traits of BC. Several previous studies have examined the correlation between CTC and patients’ clinicopathological features and have shown that CTCs were indicative for tumor burden (16, 21) or cancer developmental stages (16). We found that CTC detection rate/counts, not CTC classification, were significantly correlated to the AJCC stage and TNM T stage of BC, supporting the correlation between CTC quantity and cancer development proposed by others (16, 17). In our study, CTC detection rate and counts were found to be correlated with tumor sizes estimated by ultrasonography, but not mammography or MRI. It has been reported that ultrasonography was a more accurate predictor of tumor size, superior to clinical examination, mammography, or MRI (22). No correlation was found between CTC quantity and breast tissue composition, supporting our inference that CTCs were not associated with BC molecular subtypes. Bansal et al. used flow cytometry to examine CTC in 114 BC patients and found that CTC did not have any correlation with the tumor immunohistochemical profile (21).

We used the Cell CytoSorter^®^ platform to isolate and quantitate CTCs. Although this microfluidic-based immuno-capture platform has also been used by several other groups for BC (16, 17), its power in capturing and enumerating CTCs has not been fully evaluated. A direct comparison between Cell CytoSorter^®^ and the FDA cleared CELLSEARCH^®^ platform in detecting CTCs in BC patients is still needed.

In the context of practical applicability, CTC tests for BC screening are still in their infancy and may be several years away from broad clinical application. This technology shares two important strengths with conventional imaging modalities, high accuracy and capability of detecting early-stage BC (**Table 6**). Although CTC tests currently suffer from a major weakness of high cost, it has several advantages over conventional imaging modalities, including little dependency on medical specialists and consequentially less turnaround time (**Table 6**). The high cost of CTC tests may be significantly reduced when this technology is fully automated.

**Table 6:** Strengths and weaknesses of CTC and imaging modalities.

Tests	CTC	Ultrasonography	Mammography	MRI
**Accuracy+**	0.889	0.848	0.820	0.870
**Detecting** **early-stage BC**	Yes	Yes	Yes	Yes
**Turnaround** **Time#**				
Sample preparation/ patient preparation and imaging	5–6 h	30–60 min	10–20 min	30–60 min
Analysis and interpretation	2 h	30–60 min	30–60 min	1–2 h
Total time for reporting results	24 h	24–48 h	24–48 h	24–48 h
**Staff-dependency**				
Technician/ technical measurement	Yes	Yes	Yes	Yes
Specialist/ result interpretation	No	Yes, radiologist, >5 years medical/ radiology training	Yes, radiologist, > 10 years medical/ radiology training	Yes, radiologist, > 10 years medical/ radiology training
Cost*	High	Low	Low	Intermediate
USA (23)	NR	$90.36	$138.17	$549.71
China (24)	¥1,800–3,300	¥78.2	¥200	¥1,286.99

+Accuracy values were adopted from **Table 1**.

#Average time used at the 2nd affiliated hospital of Zhejiang University, China.

*Costs were adopted from published studies and were based on the Medicare reimbursement rate for each test in the USA and China; CTC is not covered by Medicare in China. NR: not reported by the referred study.

## Conclusions

CTC tests have a high diagnostic accuracy comparable to that of breast imaging modalities, and can be used as an alternative tool for BC screening. CTC detection rate and counts may be used as a predictor for clinicopathological changes of BC; future standardization and validation are needed.

## Data availability statement

The raw data supporting the conclusions of this article will be made available by the authors, without undue reservation.

## Ethics statement

The studies involving human participants were reviewed and approved by the ethics committee of the Second Affiliated Hospital, Zhejiang University School of Medicine (approval number, 2017-006). The patients/participants provided their written informed consent to participate in this study.

## Author contributions

Conception and design: XS and JH. Acquisition of data: XJ, ZC, KW, XY, WC, JJ, ZW, and YC. Analysis and interpretation of data: XS, ZZ, YC, W-HF, and JH. Writing of the manuscript: XS and HJ. Review of the manuscript: W-HF and ZZ: All authors have read and approved the manuscript.

## Funding

This study was partially supported by grants from Research and Application of 3D Printing Integrated Manufacturing Technology of Personalized Medical AIDS, Zhejiang Science and Technology Department (grant number 2017C01020).

## Acknowledgments

We would like to thank the Department of Radiology at the Second Affiliated Hospital of Zhejiang University for their help in medical imaging examinations. Special thanks to Dr. Yue Qu from Monash University, Australia for critically reading this manuscript and providing valuable comments.

## Conflict of interest

Authors YC and W-HF were employed by Hangzhou Watson Biotech.

The remaining authors declare that the research was conducted in the absence of any commercial or financial relationships that could be construed as a potential conflict of interest.

## Publisher’s note

All claims expressed in this article are solely those of the authors and do not necessarily represent those of their affiliated organizations, or those of the publisher, the editors and the reviewers. Any product that may be evaluated in this article, or claim that may be made by its manufacturer, is not guaranteed or endorsed by the publisher.

## Supplementary material

Supplementary Table 1 Demographic, clinical, and pathological characteristics of study participants.
