# Supplementary material for: A comprehensive comparison of circulating tumor cells and breast imaging modalities as screening tools for breast cancer in Chinese women
Source: Front Oncol. 2022 Aug 1;12:890248. doi: 10.3389/fonc.2022.890248 (PMC9377692; doi:10.3389/fonc.2022.890248)
Supplement: Supplementary Table 1 — Demographic, clinical, and pathological characteristics of study participants. [file Table_1.docx]

**Supplementary table 1: Demographic, clinical and pathological characteristics of study participants**

| **Groups** | **Breast cancer patients**  **No. (%)** | **Negative control** | |
| --- | --- | --- | --- |
|  |  | **Benign breast disease** | **Healthy women** |
|  |  |  |  |
| **Total number** | 102 | 177 | 64 |
|  |  |  |  |
| **Median age, years (range)** | 53.7 (29-75) | 43.2 (22-73) | 40.6 (21-66) |
|  |  |  |  |
| **AJCC stage** |  | Not applicable | |
| I | 42 (41.2) |  |  |
| II | 47 (46.1) |  |  |
| III | 13 (12.7) |  |  |
|  |  |  |  |
| **TNM T stage** |  |  |  |
| T1 | 64 (62.7) |  |  |
| T2 | 32 (31.4) |  |  |
| T3 | 6 (5.9) |  |  |
|  |  |  |  |
| **TNM N stage** |  |  |  |
| N0 | 62 (60.8) |  |  |
| N1 | 31 (30.4) |  |  |
| N2 | 6 (5.9) |  |  |
| N3 | 3 (2.9) |  |  |
|  |  |  |  |
| **Molecular subtypes** |  |  |  |
| Luminal A | 34 (33.3) |  |  |
| Luminal B | 36 (35.3) |  |  |
| HER2 | 12 (11.8) |  |  |
| TNBC | 20 (19.6) |  |  |
|  |  |  |  |
| **Histological grade** |  |  |  |
| 1 | 6 (5.9) |  |  |
| 2 | 40 (39.2) |  |  |
| 3 | 17 (16.7) |  |  |
